# Insectivorous birds eavesdrop on the pheromones of their prey

**DOI:** 10.1371/journal.pone.0190415

**Published:** 2018-02-07

**Authors:** Irene Saavedra, Luisa Amo

**Affiliations:** Departamento de Ecología Evolutiva, Museo Nacional de Ciencias Naturales (CSIC), C/ José Gutiérrez Abascal, Madrid, Spain; INRA-UPMC, FRANCE

## Abstract

Chemical cues play a fundamental role in mate attraction and mate choice. Lepidopteran females, such as the winter moth (*Operophtera brumata*), emit pheromones to attract males in the reproductive period. However, these chemical cues could also be eavesdropped by predators. To our knowledge, no studies have examined whether birds can detect pheromones of their prey. *O*. *brumata* adults are part of the winter diet of some insectivorous tit species, such as the great tit (*Parus major*) and blue tit (*Cyanistes caeruleus*). We performed a field experiment aimed to disentangle whether insectivorous birds can exploit the pheromones emitted by their prey for prey location. We placed artificial larvae and a dispenser on branches of Pyrenean oak trees (*Quercus pyrenaica*). In half of the trees we placed an *O*. *brumata* pheromone dispenser and in the other half we placed a control dispenser. We measured the predation rate of birds on artificial larvae. Our results show that more trees had larvae with signs of avian predation when they contained an *O*. *brumata* pheromone than when they contained a control dispenser. Furthermore, the proportion of artificial larvae with signs of avian predation was greater in trees that contained the pheromone than in control trees. Our results indicate that insectivorous birds can exploit the pheromones emitted by moth females to attract males, as a method of prey detection. These results highlight the potential use of insectivorous birds in the biological control of insect pests.

## Introduction

Chemical communication is probably the most ancient and widespread form of communication [1, 2] and plays an important role in sexual selection [3, 4]. The chemical compounds emitted by animals and used in mate attraction and mate choice are known as pheromones [4]. In many cases, the chemical signals involved in mate choice may allow potential partners to evaluate an individual´s quality. Theoretical models have predicted that signals can only be evolutionarily stable if they are condition-dependent, or costly to the signaler, and if the cost is correlated with the signaler's quality [5–7]. Therefore, individuals can not afford to cheat, i.e., to signal at too high a level [8], and conspecifics can rely on the information provided by these honest signals.

Chemical signals can provide information about the individual quality (i.e., body condition, reproductive status, age, parasite load, health condition) [4]. However, chemical signals, as well as other signals, are not only costly to produce but they may imply survival costs. Signals are not only detected by potential partners but can also be eavesdropped by predators and parasites. Therefore, the emission of chemical signals can greatly increase the risk of predation or parasitism [9, 10]. Examples of predators that eavesdrop the chemical cues involved in mate attraction or signaling have been found in different taxa, from invertebrates to vertebrates such as amphibians, reptiles and mammals [2]. For example, there are numerous studies of natural predators that are able to detect the chemical cues of their bark beetle prey [11]. Smooth snakes (*Coronella austriaca*) can also detect the chemical cues of their lizard prey [12]. Mammalian predators often detect their prey by intercepting intraspecific reproductive cues, such as least weasels (*Mustela nivalis*) [13–15], cats (*Felis catus*) and foxes (*Vulpes vulpes*) [16]. In the case of predatory birds, previous evidence suggests that some predatory species of raptors and shrikes could be visually attracted to the UV light reflected by the urine and faeces marks of their small mammal prey [17]. As some studies indicate, this detection of prey may not depend entirely on UV vision, because birds do not prefer UV areas lacking scent marks [18–20]. To our knowledge, there is no other evidence that birds use olfaction to eavesdrop the chemical signals emitted by their prey.

The lack of studies in this area is probably due to the fact that birds were considered almost anosmic in the past. However, an increasing number of studies have shown that birds can detect odors in several ecological contexts. For example, birds can use their sense of smell in intraspecific relationships [21]. The crimson rosella (*Platycercus elegans*) can discriminate between subspecies using olfaction [22]. Antarctic prions (*Pachiptila desolata*) can recognize the scent of their partners [23]. Passeriformes can discriminate the sex of conspecifics [24, 25], and Sphenisciformes, Procellariiformes and Passeriformes use olfaction for kin recognition [26, 27, 28]. Moreover, house finches (*Carpodacus mexicanus*) seem to be able to evaluate the quality of conspecifics using olfaction [29]. In interspecific contexts, blue tits (*Cyanistes caeruleus*) and European starlings (*Sturnus vulgaris*) are known to use olfaction for detecting aromatic plants [30–32]. Columbiformes and Procellariiformes use olfaction for orientation and navigation [33, 34]. For example, British storm-petrel (*Hydrobates pelagicus*) and blue petrels (*Halobaena caerulea*), can find their own burrows using olfaction [35–36]. The ability to detect the chemical cues of predators and use them to ascertain predators has been demonstrated in Passeriformes [37–39], Galliformes [40] and Anseriformes [41].

Previous evidence suggests that birds are able to perceive odors in the process of foraging. Vultures, such as turkey vultures (*Cathartes aura*) [42] and greater yellow-headed vultures (*C*. *melambrotus*) [43], appear to use olfaction to locate carcasses. The role of olfaction in foraging has also been suggested in honey-guides (family Indicatoridae) [44] and honey buzzards [45]. In addition, some species of parrots can find their food using olfaction. For example, kakapo (*Strigops habroptilus*), a flightless, nocturnal and vegetarian bird, identifies bins with food using olfaction [46]. Procellariiform seabirds use dimethyl sulphide (DMS) for foraging [47]. The DMS is produced when the zooplankton graze on the phytoplankton [47], thus signaling areas of high productivity in the oceans. By detecting this compound, Procelariiformes [36, 47] and Sphenisciformes[48, 49] can locate their prey. Insectivorous birds are also able to use olfaction to find their food. Kiwis (*Apteryx australis*) can use olfaction when foraging [50, 51]. Parids, such as great tits, can exploit the herbivore-induced volatiles that trees emit in response to lepidopteran caterpillar infestation for finding those caterpillars upon which they prey [52]. Attraction to trees infested with caterpillars has also been shown in different plant-insect-bird systems [53–57].

Birds may not only use indirect cues to find their prey [47, 52, 56], but may be able to detect the chemical cues emitted by the prey itself. In many lepidopteran species, females release pheromones during the reproductive period in order to attract males [58, 59]. Birds could detect these pheromones and use them to locate their prey. In this way, they could maximize their foraging effort. However, to our knowledge, no study has examined whether insectivorous birds can use olfaction to detect the pheromones of adult lepidopteran. Therefore, the aim of this study was to analyze whether insectivorous birds can eavesdrop on the sex pheromones of lepidoptera females. We performed a field experiment to investigate whether insectivorous birds in the wild are attracted to the pheromones of one of their potential prey, *O*. *brumata* adults. The winter moth is considered a plague in many forests and orchards in Europe [60]. We measured whether the predation rates on artificial larvae located in Pyrenean oak trees containing an *O*. *brumata* pheromone dispenser differed from those containing a control dispenser. We expected that if birds can detect the pheromones of their prey and are attracted to them, the predation rate of artificial larvae by birds will be higher in the trees that contain a pheromone dispenser than in the trees that contain a control dispenser.

## Materials and methods

### Study area and species

The experimental study was carried out between May and June 2016 in a Pyrenean oak (*Quercus pyrenaica*) forest included in a Site of Community Interest (SCI), located in Sierra de Fonfría, in Teruel province, Spain (40°59′N, 1°05′W). In this forest, a population of insectivorous birds breeding in 100 wooden nest-boxes was established in 2011. Nest-boxes were occupied mainly by breeding pairs of blue tits (*Cyanistes caeruleus*) (around 45 pairs), and some pairs of great tits (*Parus major*) (around 10 pairs). Other insectivorous bird species were observed in the study area at lower densities, including common blackbird (*Turdus merula*), Eurasian blackcap (*Sylvia atricapilla*), Sardinian warbler (*S*. *melanocephala*) and common nightingale (*Luscinia megarhynchos*). Tits feed mainly on caterpillars, such as the *O*. *brumata*, during the breeding period [61, 62]. However, during the winter, when no caterpillars are available, parids like great tits and blue tits prey upon *O*. *brumata* adults [61, 63]. Thus, *O*. *brumata* adults constitute an important part of the diet when they are available in winter. Others species included in the winter diet of tits belong to the Hemiptera, Lepidoptera, Coleoptera and Hymenoptera orders [61]. *O*. *brumata* adults are present in the study area from November to February [64]. In this species only females produce pheromones during the reproductive period to attract males [65, 66]. In 1982, the pheromone of *O*. *brumata* was identified as 1,Z3,Z6,Z9-nonadecatetraene [65, 66]. A synthetic pheromone can be obtained from commercial supplier (ControlBio® from OPENNATUR, S.L.). The pheromone dispensers attached to a trap are effectively used in insect pest control in order to reduce male quantities. The pheromone dispensers contain 0.5 mg of 1,Z3,Z6,Z9-nonadecatetraene (Pherobank, B.V.). The emission lasts 40 days, and thus the emission rate is approximately 9 ng/min (Pherobank, B.V.). We performed the study outside of the reproductive period of this species to ensure that adult moths were absent, and therefore, bird attraction to the *O*. *brumata* pheromone could be attributed to the pheromone and not to the presence of males.

### Experimental design and procedure

**Fig 1:**
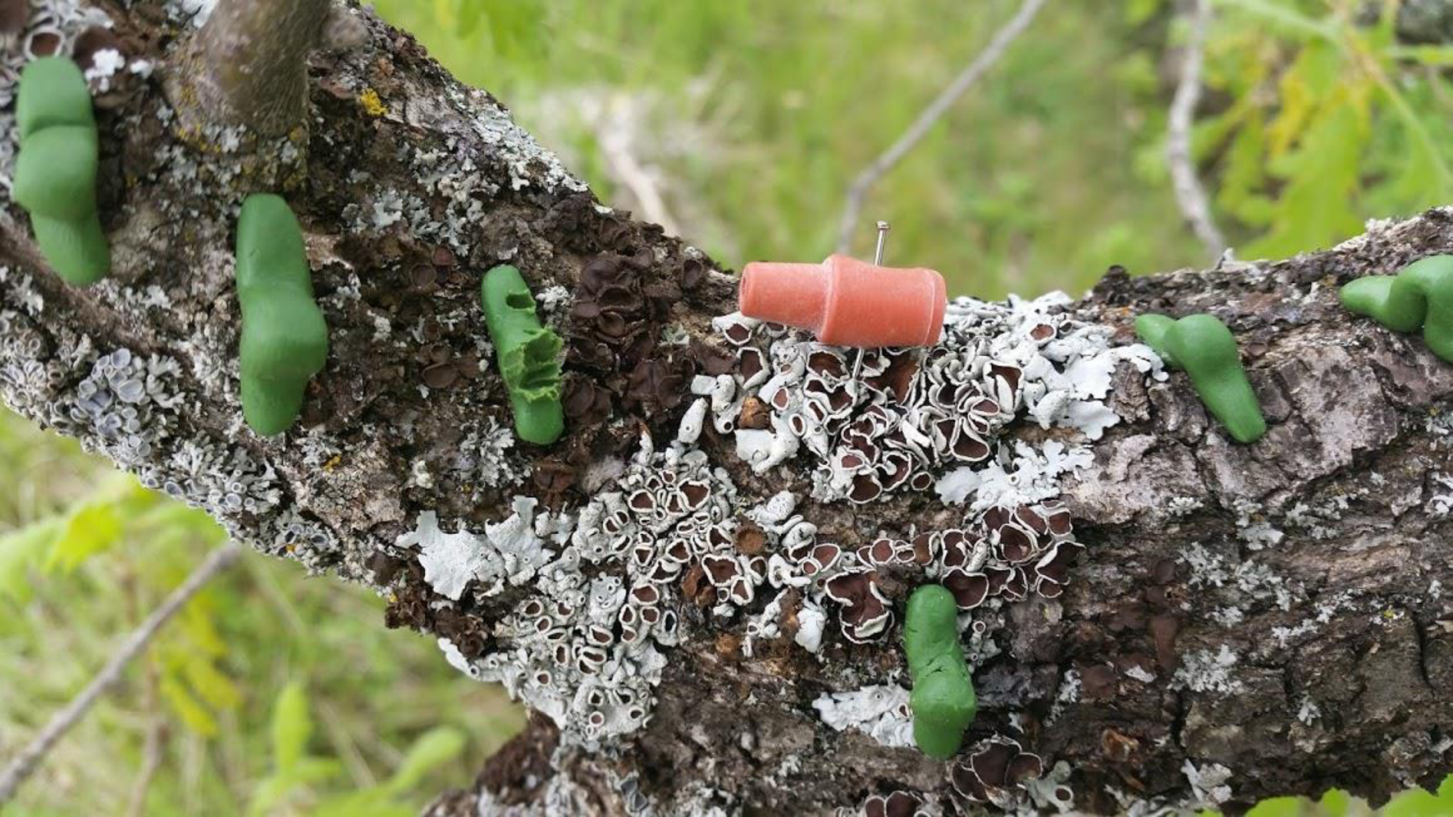
Photograph of the pheromone dispenser and several plasticine caterpillars, one of them with beak marks, indicating a predation event by an insectivorous bird.

*O*. *brumata* adults can only be found in winter. Nevertheless, one week before the experiment, we placed 5 moth traps with commercial *O*. *brumata* pheromone for one week in different locations in the study area to ensure there were no *O*. *brumata* adults or other insect species that could have been attracted by the same pheromone (e.g., predators or parasitoids). We found no *O*. *brumata* adults or other arthropods inside the traps. During the 27 days of the experiment, we placed one dispenser and ten artificial larvae on branches of 32 Pyrenean oak trees. The branches were approximately 1.5 m long with no evident signs of herbivory. The dispenser and artificial larvae were placed at similar average heights in the trees (approx. 1.5 m high). Dispensers were fixed to the branch with a pin. Ten artificial larvae were placed in the surroundings of the dispenser, from 2 to 50 cm from the dispenser. Thus, dispensers were situated in the middle of ten artificial larvae. Pheromone and control dispensers were brown and opaque (approx. 20x10 mm). Pheromone dispensers were made with natural rubber (Fig 1). Control dispensers were made of brown plasticine similar to the color of the pheromone dispenser. There were no significant differences between the reflectance spectra of the two types of dispensers (p > 0.05; see Fig A in S1 Supporting Information).

We selected trees that were located within 10 meters from a nest-box, and therefore, within the breeding territory of a blue tit or a great tit. Thirty-one of the 32 nest-boxes close to experimental trees were occupied by blue tits. The artificial larvae were made of light green plasticine (similar to the natural color of real *O*. *brumata* larvae, at least by human-visual perception). Neither the plasticine caterpillars nor the dispensers emitted UV light. The plasticine larvae were approximately the size of a large fifth instar *O*. *brumata* larva (length 25–30 mm, Ø 3–4 mm). The plasticine larvae were attached with cyanoacrylate adhesive glue on branches of 32 forest oak trees. Experimental trees were separated by at least 40 meters. The trees were alternatively assigned to one of the treatments: commercial *O*. *brumata* pheromone dispenser (n = 16), or plasticine dispenser, simulating the shape of the commercial pheromone dispenser (odorless control) (n = 16). Thus, treatments were spatially inter-mixed in the oak trees.

To study the attraction of the insectivorous birds to the *O*. *brumata* pheromone, we checked the number of larvae with predation marks by birds in the trees. Artificial caterpillar models have previously been used to estimate insectivorous bird attraction [54, 67–72]. A predation event was assigned to a tree when the tree contained at least one larva damaged by birds. Larva models were considered damaged when they had triangle-shaped marks and deep cuts made by the beak of the birds and when a part of their body was taken by the birds, as described in Mäntylä and collaborators [54, 69]. From the following day onwards we checked the condition of these plasticine larvae every two days for the first ten days. After ten days, we checked them twice, once at day 20 and again at day 27 from the beginning of the experiment. Each model showing a predation mark was replaced with a new one at the same location during the visits. The treatments were in place for 27 days, a period of time for which the effectiveness of the commercial pheromone is guaranteed, as it can last up to 40 days (Pherobank, B.V.). At the end of the experiment, we removed all plasticine larvae and the commercial pheromones and controls. The experiment was conducted under a license issued by the Instituto Aragonés de Gestión Ambiental (INAGA/500201/24/2015/11696).

### Statistical analyses

We modeled the probability that at least one predation event occurs in a tree in relation to the treatment (pheromone vs control) with a generalized linear model (GLM) fit by the Laplace approximation with binomial errors and a logit link function. We also analyzed the probability that the proportion of damaged larvae per tree differed in relation to the treatment with a generalized linear model (GLM) fit by the Bernoulli distribution with binomial errors and a logit link function. We included the day of observation in the initial models but, as it was not significant (see S2 Supporting Information), it was removed from the final models. Data analyses were performed with the statistical program R 2.15.1 “stats” package [73].

## Results

**Fig 2:**
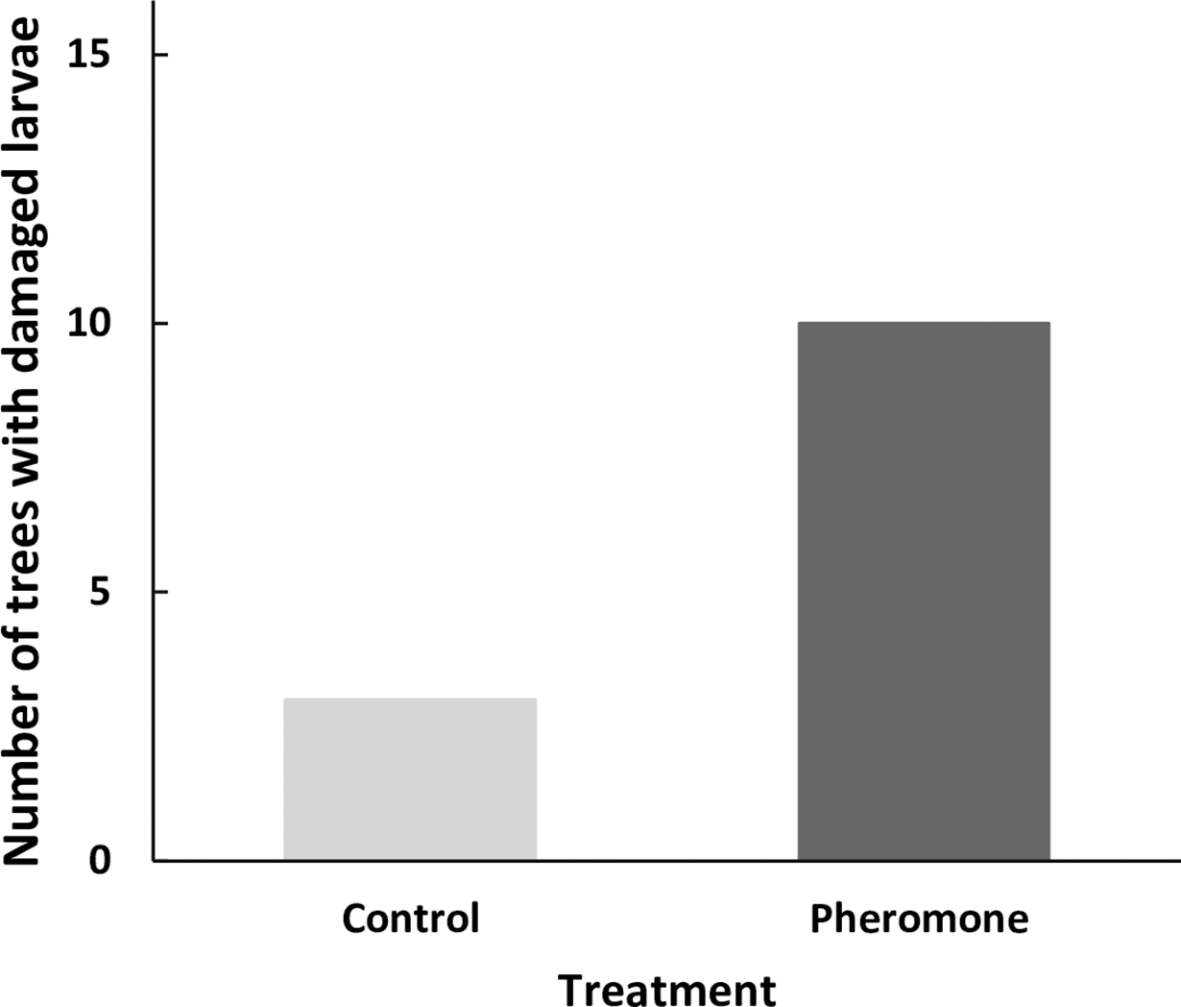
Number of trees that had at least one artificial larva with marks of avian predation when the tree contained an *Operophtera brumata* pheromone dispenser and when the tree contained a control dispenser.

The number of trees that had at least one caterpillar with signs of avian predation differed between treatments (GLM: *Z* = 2.40, *P* = 0.02, Fig 2). Ten out of the 16 trees containing a pheromone dispenser had at least one avian predation event (i.e., at least one artificial caterpillar had signs of avian predation, Fig 1). In contrast, a predation event was observed in only 3 out of the 16 control trees. The proportion of larvae damaged by the birds differed between treatments (GLM: *Z* = -3.72, *P* = 0.0002; see Table A in S3 Supporting Information), being significantly higher in trees that contained a pheromone (Mean ± SE = 3.04% ± 1.48%) than in control trees (Mean ± SE = 0.71% ± 0.54%).

## Discussion

Our results show for the first time that insectivorous birds can exploit sex pheromones for prey location [74]. A greater number of trees were visited by birds (i.e., they had at least one artificial caterpillar with signs of avian predation) when they contained an *O*. *brumata* pheromone dispenser compared to a control dispenser (Fig 2). Additionally, a greater proportion of artificial larvae were predated when the tree in which they were located contained an *O*. *brumata* pheromone dispenser than when it contained a control dispenser.

We performed the study during the spring, when there are no adults of *O*. *brumata*. The adults of this species emerge in November and can be observed in the field until February [64]. Therefore, when we placed the pheromones in the field, the attraction of males to this pheromone was not possible. Synthetic pheromones can be less specific that the natural female pheromones. Thus, we investigated the presence of adults in the study area to examine whether the pheromone attracts other insects. We placed five traps containing the pheromone a week before the beginning of the experiment and no moths or other arthropods were collected. Other arthropod species were never observed close to dispensers or artificial larvae in the study area. Moreover, we did not observe any damage to the caterpillars due to species other than birds. Therefore, the greater predation rate of artificial larvae does not appear to be due to the attraction of birds to the presence of *O*. *brumata* males or other arthropods close to the female pheromone.

Control and pheromone dispensers were made of different material, which may have induced differences in visual and odor cues available to birds between control and treated dispensers. It is, however, unlikely that visual cues account for the differences in predation rates because the color spectra of control and pheromone dispensers were not significantly different (see Figure A in S1 Supporting Information). Therefore, the lack of significant differences between dispensers in visual cues eliminates the possibility that birds were attracted to the dispenser's appearance. The artificial caterpillars and control dispensers were made with plasticine, and thus the volatiles emitted by plasticine would be present in both treatments. The similarity in the chemical composition between the caterpillars and the control dispensers would mean that bird attraction was not caused by differences in the volatiles emitted by the different materials of the two types of dispensers. Regardless, further experiments using the same dispensers in the control and pheromone treatments are needed to completely exclude the possibility of an artifact. The emission rate of the pheromone dispenser was around 9 ng/min. This emission rate is higher than that produced by a single female moth [75], and may be similar to that produced by 10 females. However, the concentration of this synthetic pheromone attracts male moths (L. Amo, personal observation), suggesting that the emission rate of the pheromone dispensers may be biologically relevant for male moths. Our results now show that vertebrate predators, such as insectivorous birds, are also attracted to the emission rate of this pheromone dispenser. However, birds could only detect female moths when the emission rate is 10-times higher than that of a single female. Thus, further studies will test whether birds can detect lower concentrations of the pheromone to disentangle whether birds can use the pheromone emissions to locate a single female moth or whether they are only attracted to female groups. Furthermore, female moths become active from sunset, when diurnal insectivorous birds such as blue tits decrease their foraging activity and search for roosting places. Therefore, additional studies are needed to elucidate whether birds use pheromones as a precise localization cue or to find good areas for foraging the subsequent day.

Previous studies have shown that the use of artificial caterpillars is a reliable measure of insectivorous bird attraction [e.g. 54, 57, 69, 72]. For example, Muiruri and collaborators observed several individuals of breeding bird species pecking the artificial caterpillars [76]. To prevent the same bird from visiting all the trees, we placed the caterpillars in trees that were separated by at least 40 meters. Blue tits normally feed within 20 meters from the nest-box in deciduous forests and up to 40 meters in mixed forests [77]. Additionally, results of another study show that 90% of foraging observations of blue and great tits were made within 45 m from the nest-box [78]. Therefore, it is unlikely that the same individuals visited more than one experimental tree. Furthermore, we expected that birds learned to recognize plasticine caterpillars as unpalatable, unprofitable prey. Thus, we did not expect birds to return to the same location numerous times.

The study area has had a blue and great tit population breeding in nest-boxes since 2011. Blue and great tits are the most abundant insectivorous birds in the area (more than 100 adults and their nestlings) [79]. The high abundance of these two species, as well as the fact that experimental trees were close to nest-boxes, suggest that these two species were likely responsible for the majority of the predation. Nonetheless, we cannot exclude the possibility that other insectivorous species or even omnivorous species that include moths in their diets and are present in the study area, may have been attracted to the pheromones.

Our results show that birds can exploit the pheromones emitted by *O*. *brumata* females. The attraction of birds to the pheromones of this species may help birds maximize their foraging effort. *O*. *brumata* females are wingless [80] and their brown coloration allows them to blend into the trunks of trees, such as the Pyrenean oak, which could make them cryptic and probably hampers visual detection by bird predators. Therefore, by using the chemical cues emitted by female moths, birds can enhance their probability of finding the camouflaged females, as well as the male moths that are attracted by the female pheromone. As a consequence, males may also suffer an increased risk of predation [10].

Bird predation on *O*. *brumata* adults during the winter may reduce the number of lepidopteran clutches and therefore, the number of caterpillars in spring. This can have important consequences for host trees in the subsequent spring. Previous studies using bird exclusion have shown a positive effect of birds on predation rates of arthropods in spring or summer [81]. However, the effect of avian predation on their prey population during the winter period has been less studied [60, 82, 83].

Insectivorous birds are predators of lepidopteran moths, eggs and caterpillars, such as the *O*. *brumata* [61, 84]. Thus, they may not only decrease the number of moth adults during the winter, but also caterpillar numbers during the spring. The nestling period of many insectivorous bird species coincides with the peak occurrence of most caterpillars, including the *O*. *brumata* larva. Thus, birds can greatly reduce the number of lepidopteran larvae feeding on trees [62]. Insectivorous birds, at least great tits, use olfaction to discriminate between trees infested with *O*. *brumata* caterpillars and uninfested trees [52], thanks to the herbivore-induced volatiles (HIPVs) that trees emit in response to herbivory [52]. The attraction of birds to caterpillar infested trees can decrease herbivore damage to trees [62, 85, 86], leading to increased growth and reduced mortality of the trees [86–88].

The attraction of birds to the pheromones of moth females adds birds to the list of predators that are able to eavesdrop the chemical cues emitted by their prey for mate attraction. This new evidence indicates the costs of the emission of chemical signals for females [89] as well as the costs of responding to such chemicals for males [10]. These results indicate the potential use of insectivorous birds in controlling Lepidopteran numbers in forests and orchards. Traditional control of adult numbers is based on the use of pheromone traps to collect males and remove them from the population, decreasing access to males by females, and therefore decreasing fecundity of females [90]. However, birds prey upon both females and males and may be much more efficient than pheromone traps in decreasing the number of adults.

## Supporting information

S1 Supporting Information **Fig A.** Reflectance spectra of control (blue line) and pheromone (brown line) dispensers.
(PDF)

S2 Supporting Information Statistical analysis of data including the day of observation in the initial models. (PDF)

S3 Supporting Information **Table A.** Number of damaged and undamaged larvae in the control and pheromone treatment.
(PDF)
