# Supplementary material for: Insectivorous birds eavesdrop on the pheromones of their prey
Source: PLoS One. 2018 Feb 7;13(2):e0190415. doi: 10.1371/journal.pone.0190415 (PMC5802436; doi:10.1371/journal.pone.0190415)

S1 Supporting Information

Fig A. Reflectance spectra of control (blue line) and pheromone (brown line) dispensers. There were not significant differences between the spectra of both dispensers ($F_{1,9}$ = 128.28, $p > 0.05$).

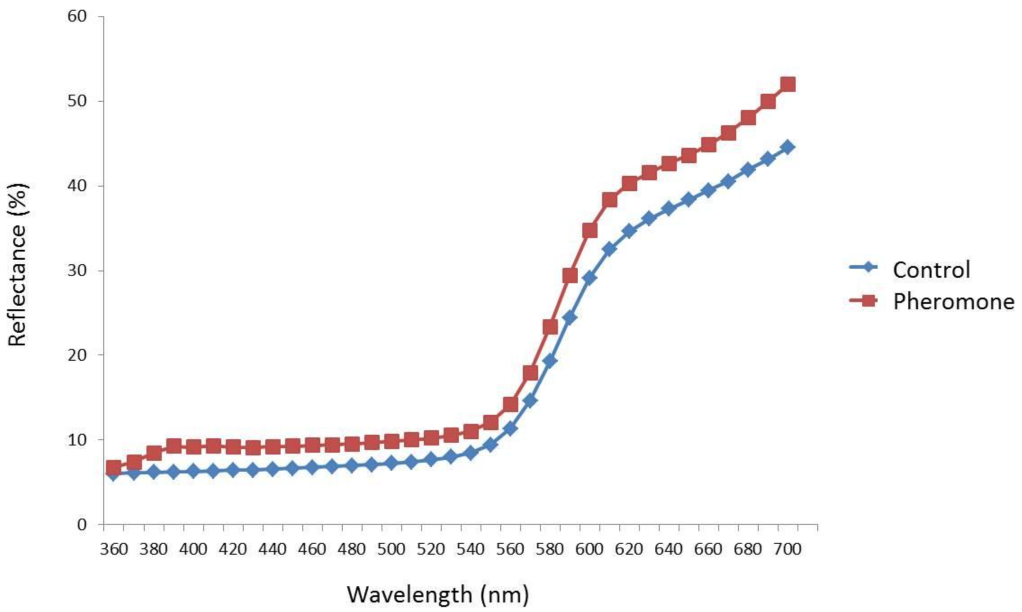

Supplement: S1 Supporting Information — Fig A. Reflectance spectra of control (blue line) and pheromone (brown line) dispensers. (PDF) [file pone.0190415.s001.pdf]
