# Supplementary material for: Insectivorous birds eavesdrop on the pheromones of their prey
Source: PLoS One. 2018 Feb 7;13(2):e0190415. doi: 10.1371/journal.pone.0190415 (PMC5802436; doi:10.1371/journal.pone.0190415)
Supplement: S2 Supporting Information — (PDF) [file pone.0190415.s002.pdf]

S2 Supporting Information

Statistical analysis of data including the day of observation in the initial models

We modelled the probability that at least one predation event occurs in a tree in relation to the treatment and day of observation with a generalized linear mixed model (GLMM) fit by the Laplace approximation with binomial errors and a logit link function. We included the tree as a random factor. We also used a GLMM to analyze the number of caterpillars attacked by birds in relation to the treatment and the day of observation (fixed factors). The tree was included as a random factor.

Results

The number of trees that had at least one caterpillar with signs of avian predation differed between treatments (GLMM: $Z = -2.08$, $P = 0.04$) but did not differ in relation to the day of observation (GLMM: $Z=-0.09$, $p=0.93$).

There were differences between treatments in the number of damaged caterpillars (GLMM: $Z = -2.17$, $P = 0.03$). The day of observation did not influence the number of caterpillars that were attacked by birds (GLMM: $Z = 1.75$, $P = 0.08$).
