# Supplementary material for: Insectivorous birds eavesdrop on the pheromones of their prey
Source: PLoS One. 2018 Feb 7;13(2):e0190415. doi: 10.1371/journal.pone.0190415 (PMC5802436; doi:10.1371/journal.pone.0190415)
Supplement: S3 Supporting Information — Table A. Number of damaged and undamaged larvae in the control and pheromone treatment. (PDF) [file pone.0190415.s003.pdf]

S3 Supporting Information

Table A. Number of damaged and undamaged larvae in the control and pheromone treatment.

**Treatment**	**damaged larvae**	**undamaged larvae**
control	0	70
control	0	70
control	0	70
control	0	70
control	6	64
control	0	70
control	0	70
control	0	70
control	0	70
control	0	70
control	0	70
control	0	70
control	1	69
control	0	70
control	1	69
control	0	70
pheromone	3	67
pheromone	0	70
pheromone	1	69
pheromone	1	69
pheromone	0	70
pheromone	17	53
pheromone	2	68
pheromone	2	68
pheromone	0	70
pheromone	1	69
pheromone	0	70
pheromone	0	70
pheromone	0	70
pheromone	4	66
pheromone	2	68
pheromone	1	69
